# Analysis of α-synuclein species enriched from cerebral cortex of humans with sporadic dementia with Lewy bodies

**DOI:** 10.1093/braincomms/fcaa010

**Published:** 2020-02-11

**Authors:** John B Sanderson, Suman De, Haiyang Jiang, Matteo Rovere, Ming Jin, Ludovica Zaccagnini, Aurelia Hays Watson, Laura De Boni, Valentina N Lagomarsino, Tracy L Young-Pearse, Xinyue Liu, Thomas C Pochapsky, Bradley T Hyman, Dennis W Dickson, David Klenerman, Dennis J Selkoe, Tim Bartels

**Affiliations:** f1 Ann Romney Center for Neurologic Diseases, Department of Neurology, Brigham and Women’s Hospital, Harvard Medical School, Boston, MA 02115, USA; f2 Department of Chemistry, University of Cambridge, Cambridge CB2 1EW, UK; f3 UK Dementia Research Institute, Department of Chemistry, University of Cambridge, Cambridge CB2 0AH, UK; f4 UK Dementia Research Institute, Department of Neurology, University College London, London WC1E 6BT, UK; f5 Department of Chemistry, Rosenstiel Institute for Basic Biomedical Research, Brandeis University, Waltham, MA 02453, USA; f6 Massachusetts General Hospital, Harvard Medical School, Department of Neurology, Massachusetts Institute for Neurodegenerative Disease, Boston, MA 02129, USA; f7 Neuropathology Laboratory, Department of Neuroscience, Mayo Clinic Jacksonville, Jacksonville, FL 32224, USA

**Keywords:** α-synuclein, Lewy body dementia, neurotoxicity, human tissue

## Abstract

Since researchers identified α-synuclein as the principal component of Lewy bodies and Lewy neurites, studies have suggested that it plays a causative role in the pathogenesis of dementia with Lewy bodies and other ‘synucleinopathies’. While α-synuclein dyshomeostasis likely contributes to the neurodegeneration associated with the synucleinopathies, few direct biochemical analyses of α-synuclein from diseased human brain tissue currently exist. In this study, we analysed sequential protein extracts from a substantial number of patients with neuropathological diagnoses of dementia with Lewy bodies and corresponding controls, detecting a shift of cytosolic and membrane-bound physiological α-synuclein to highly aggregated forms. We then fractionated aqueous extracts (cytosol) from cerebral cortex using non-denaturing methods to search for soluble, disease-associated high molecular weight species potentially associated with toxicity. We applied these fractions and corresponding insoluble fractions containing Lewy-type aggregates to several reporter assays to determine their bioactivity and cytotoxicity. Ultimately, high molecular weight cytosolic fractions enhances phospholipid membrane permeability, while insoluble, Lewy-associated fractions induced morphological changes in the neurites of human stem cell-derived neurons. While the concentrations of soluble, high molecular weight α-synuclein were only slightly elevated in brains of dementia with Lewy bodies patients compared to healthy, age-matched controls, these observations suggest that a small subset of soluble α-synuclein aggregates in the brain may drive early pathogenic effects, while Lewy body-associated α-synuclein can drive neurotoxicity.

## Teaser

Previous work suggests the existence of soluble, diffusible α-synuclein species in the brains of synucleinopathy patients. In this study, we show that soluble, non-physiological α-synuclein in dementia with Lewy bodies brains permeabilize lipid membranes *in vitro* compared with identically prepared extracts from control brains. In addition, we show that Lewy-associated species from these brains are neurotoxic.

## Graphical Abstract

**Graphical Abstract:**
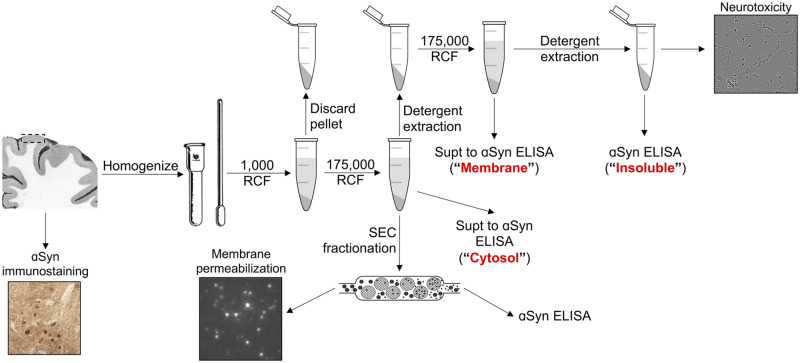

See Castillo-Carranza (https://doi.org/10.1093/braincomms/fcaa016) for a scientific commentary on this article.

## Introduction

Dementia with Lewy bodies (DLB) is an aggressive neurodegenerative disease associated with progressive cognitive dysfunction and hallucinations. Neuropathologically, the presence of intraneuronal aggregates of α-synuclein (αSyn), similar to those of Parkinson’s disease (PD), is diagnostic (McKeith *et al.*, 1996; Spillantini *et al*., 1997; McKeith *et al.*, 1999; McKeith *et al.*, 2005; McKeith *et al.*, 2017). In both PD and DLB, αSyn forms hallmark perikaryal and neuritic aggregates called Lewy bodies and Lewy neurites, in addition to more recently described small synaptic aggregates (Kramer and Schulz-Schaeffer, 2007; Roberts *et al.*, 2015).

αSyn is a 140-amino-acid intracellular protein with a physiological role in regulating the trafficking of synaptic and other small vesicles (Cabin *et al.*, 2002; Burré *et al.*, 2010; Sharma *et* *al*., 2011; Burré *et al.*, 2014; Logan *et al.*, 2017). To date, six different point mutations within the *SNCA* gene have been linked to early-onset PD and/or DLB, indicating that αSyn abnormalities can be causative in the pathogenesis of these diseases (Polymeropoulos *et al.*, 1997; Krüger *et al.*, 1998; Singleton *et al.*, 2003; Chartier-Harlin *et al.*, 2004; Zarranz *et al.*, 2004; Appel-Cresswell *et al.*, 2013, Lesage *et al.*, 2013; Pasanen *et al.*, 2014).

Despite the genetic and neuropathological evidence suggesting a critical role for αSyn in DLB and PD pathogenesis, the mechanism by which αSyn leads to widespread neurodegeneration remains poorly understood. The evidence in favour of the intrinsic toxicity of Lewy bodies is conflicting (Calne and Mizuno, 2004; Tanaka *et al.*, 2004; Greffard *et al.*, 2010; Osterberg *et al.*, 2015; Power *et al.*, 2017). This discordance suggests that the highly insoluble, presumably filamentous αSyn in Lewy bodies may not be the principal neurotoxic species underlying clinical symptoms. A large body of work, predominantly assessing recombinant αSyn, indicates that soluble oligomers show neurotoxic properties, including a propensity to alter cellular membranes (Volles *et al.*, 2001; Putcha *et al.*, 2010; Fusco *et al.*, 2016). Validation of these *in vitro* results in human tissue is incomplete, however. Regional staging of αSyn aggregates in synucleinopathy patients suggests a spatiotemporal progression of pathology (Marui *et al.*, 2002; Braak *et al.*, 2004; Deramecourt *et al.*, 2006; Del Tredici and Braak, 2015), potentially mediated by similar diffusible αSyn species. In fact, αSyn-containing insoluble extracts from human synucleinopathy brain tissue can propagate the aggregation of endogenous αSyn in cellular models (Prusiner *et al.*, 2015; Woerman *et al.*, 2015; Cavaliere *et al.*, 2017). In addition, previous *in vivo* and *in vitro* studies suggest the existence of a soluble, diffusible αSyn species that might cause the spreading of Lewy pathology and neurotoxicity (Volpicelli-Daley *et al.*, 2011; Luk *et al.*, 2012; Kovacs *et al.*, 2014; Volpicelli-Daley *et al.*, 2014; Xin *et al.*, 2015; Osterberg *et al.*, 2015, Mason *et al.*, 2016; Rey *et al.*, 2016; Sacino *et al*., 2016; Blumenstock *et al.*, 2017; Peng *et al.*, 2018). Clinically, ‘post-mortem’ analyses of PD patients who had received dopaminergic neuron grafts showed αSyn pathology (Kordower *et al.*, 2008; Li *et al.*, 2008), although this phenomenon was not observed in all neural graft recipients (Mendez *et al.*, 2008), nor does it explain the selective vulnerability of the respective neuronal populations observed in synucleinopathies (Walsh and Selkoe, 2016).

Some work does begin to characterize these potentially toxic αSyn species. For example, application of oligomer-selective antibodies (Paleologou *et al.*, 2009; Kovacs *et al.*, 2012) or single-chain antibody fragments (Emadi *et al.*, 2007; Emadi *et al.*, 2009; Xin *et al.*, 2015) to soluble fractions of brain homogenates demonstrates the likely presence of misfolded oligomeric αSyn species that are distinct from the physiological multimeric αSyn that exist in complex equilibrium with monomeric αSyn (Mor *et al*., 2016; Bartels *et al.*, 2011; Wang *et al.*, 2011; Burré *et al.*, 2014; Wang *et al.*, 2014; Luth *et al.*, 2015). Similarly, the use of a proximity ligation assay on fixed brain tissue sections of synucleinopathy patients revealed abundant oligomeric αSyn species previously undetectable by traditional immunostaining methods (Kramer and Schulz-Schaeffer, 2007; Roberts *et al.*, 2015). Other work has shown vesicle-associated, soluble αSyn oligomers associated with DLB (Sharon *et al.*, 2003).

To enrich and further characterize oligomeric αSyn from DLB patients, we obtained tissue from numerous *post mortem* brains with a neuropathological diagnosis of DLB, histochemically confirmed the presence αSyn Lewy-type αSyn neuronal inclusions in this tissue, and observed substantial levels of detergent-insoluble αSyn, as has previously been reported (Campbell *et al.*, 2000; Klucken *et al.*, 2006). Fractionation of the brain tissue extracts by ultracentrifugation and size-exclusion chromatography (SEC) yielded buffer-soluble fractions separated by molecular radius. We quantified buffer-soluble, high molecular weight (HMW) αSyn and then searched for specific neurotoxicity of these fractions compared with simultaneously prepared cytosolic fractions of non-neurodegenerative control brains. Given previous *in vitro* studies showing the ability of αSyn to permeabilize membranes (Stefanovic *et al*., 2015; Di Scala *et al*., 2016; Ludtmann *et al.*, 2018), we then tested the ability of these enriched species to permeabilize lipid vesicles in a recently developed *in vitro* assay (Flagmeier *et al.*, 2017). In addition to testing these soluble species, we analysed detergent-insoluble material from the same brains for neurotoxicity to elucidate the potential mode of bioactivity of Lewy body (LB)-associated αSyn. This study provides evidence from human samples to support previous *in vitro* and animal-based work on the role of αSyn in synucleinopathy pathogenesis.

## Materials and methods

### Human tissue

Human brain tissue was provided by Brigham and Women’s Hospital (Boston, MA, USA), Mayo Clinic (Jacksonville, FL, USA), Massachusetts General Hospital/Massachusetts Alzheimer’s Disease Research Center (Boston, MA, USA), Newcastle Brain Tissue Resource (Newcastle upon Tyne, UK) and Queen Square Brain Bank for Neurological Disorders (London, UK). Information about the brain samples used in this study is summarized in Table 1. Consent was obtained from patients prior to death at each brain collection centre. All five brain banks approved of the proposal for the use of human tissue in this study, and the IRB at the first and last authors’ institution deemed the planned use of this tissue to be appropriate and ethical.

**Table 1:** Tissue samples were collected from five separate sources: Brigham and Women’s Hospital (Boston, MA, USA); Mayo Clinic (Jacksonville, FL, USA); Massachusetts General Hospital/Massachusetts Alzheimer’s Disease Research Center (Boston, MA, USA); Newcastle Brain Tissue Resource (Newcastle upon Tyne, UK); and Queen Square Brain Bank for Neurological Disorders (London, UK)

Case #	Source	Diagnosis	Age	Sex	Duration of disease (years)	PMI (h)
20060025	Newcastle	Probable DLB	76	M	8	13
20050022	Newcastle	PD with DLB	68	F	9	69
20070105	Newcastle	Probable DLB	71	M	7	8
20040085	Newcastle	Probable DLB	77	M	2	29
20100353	Newcastle	Cog. normal control	74	F	NA	53
20100150	Newcastle	Cog. normal control	77	M	NA	83
A01-064	Brigham and Women's	Interstitial pneumonitis	49	F	NA	25.5
A01-111	Brigham and Women's	Cog. normal control	43	F	NA	23.5
A01-213	Brigham and Women's	DLB	83	M	8	54
BN14D00074	Brigham and Women's	DLB	82	M	5	<24
P80/10	Queen Square	DLB	67	M	7	40
P11/11	Queen Square	DLB	60	M	8	24
P20/14	Queen Square	DLB	75	M	16	57
P48/07	Queen Square	Cog. normal control		F	NA	39
1687	MGH	DLB	73	M		24
1650	MGH	DLB	76	M		10
1594	MGH	DLB	81	F		8
1590	MGH	DLB	62	M		<24
1504	MGH	DLB	79	F		12
1751	MGH	DLB	87	F		6
1901	MGH	Control	54	M	NA	6
1887	MGH	Control	60	M	NA	14
CON 1	Mayo Clinic	PA/CAA	81	F	NA	
CON 2	Mayo Clinic	PA	75	F	NA	
CON 3	Mayo Clinic	PA	81	F	NA	22
CON 4	Mayo Clinic	PA/lacune (pseudo-dementia)	75	F	NA	
CON 5	Mayo Clinic	PA	81	M	NA	
CON 6	Mayo Clinic	PA	78	M	NA	13
CON 7	Mayo Clinic	SC/VaD	79	F	NA	8
CON 8	Mayo Clinic	SC	70	M	NA	20
CON 9	Mayo Clinic	Normal (pseudo-dementia)	63	M	NA	3
CON 10	Mayo Clinic	PA	87	F	NA	21
CON 11	Mayo Clinic	PA	88	F	NA	6
Syn1	Mayo Clinic	DLB	81	F		
Syn2	Mayo Clinic	DLB	67	F		
Syn3	Mayo Clinic	DLB	68	F	11	
Syn4	Mayo Clinic	DLB	66	F		16
Syn5	Mayo Clinic	DLB	79	M		
Syn6	Mayo Clinic	DLB	54	M		5
Syn7	Mayo Clinic	DLB	62	F		7

Frontal cortex grey matter was analysed from DLB patients and corresponding controls.

CAA: cerebral amyloid angiopathy; F: female; M: male; MGH: Massachusetts General Hospital; NA: not applicable; PA: pathological aging; PMI: post-mortem interval; SC: senile changes; VaD: vascular dementia.

### Sequential extraction of human tissue

Frontal cortex tissue pieces weighing between 250 and 600 mg were Dounce homogenized with 20 strokes at 2500 rpm using an overhead stirrer (Wheaton, Millville, NJ, USA) in four volumes (weight:volume) tris-buffered saline (TBS)/protease inhibitor (PI) (20 mM Tris–HCl, 500 mM NaCl, pH 7.5 with complete PI tablet; Sigma-Aldrich, St. Louis, MO, USA). Homogenates were centrifuged for 5 min at 1000 × *g* at 4°C to remove highly insoluble structures and debris from the tissue. The resulting supernatants were centrifuged for 30 min at 175 000 × *g*. The high-speed supernatant was collected as ‘cytosol’, and a small aliquot was saved for enzyme-linked immunosorbent assay (ELISA) αSyn quantification. The remaining supernatant was flash-frozen in liquid nitrogen and stored at −80°C until SEC fractionation. The resulting pellet was resuspended in either 1% Triton X-100 (TX) in TBS or in modified radioimmunoprecipitation assay (OG-RIPA) buffer [0.5% Nonidet P-40 substitute, 0.5% sodium deoxycholate, 0.1% sodium dodecyl sulphate (SDS), 10 mM calcium chloride with 2% *n*-octyl-β-d-glucoside; Abcam, Cambridge, MA, USA] (Kan *et al.*, 2013) and sonicated to homogeneity using a Dismembrator 300 Microtip Sonicator (Fisher Scientific, Hampton, NH, USA) set to 40% output. This homogenate was centrifuged for 30 min at 175 000 × *g* at 4°C. The resulting OG-RIPA supernatants were collected, and the pellets were resuspended in 8 M urea with 5% SDS in TBS and boiled for 10 min at 100°C. The resulting TX supernatants were collected, and the remaining pellets were resuspended in 5% SDS in TBS, sonicated to homogeneity and centrifuged for 30 min at 175 000 × *g* at 4°C. The resulting SDS pellets were resuspended in 8 M urea with 5% SDS in TBS and boiled for 10 min at 100°C.

### Comparison of membrane-associated α-synuclein from different extraction methods

To analyse membrane-associated αSyn levels, the 1% Triton X-100 and 2% SDS extracts from our original extraction protocol were pooled and compared with the OG-RIPA extracts generated from our final quantitative extraction method (Supplementary Fig. 1). Both methods extract similar levels of αSyn by the percentage of total, although extraction by OG-RIPA instead of TX and SDS yielded higher levels of membrane-associated αSyn at the expense of Lewy-associated αSyn in terms of αSyn amount normalized to total protein.

### Antibodies

2F12 and SOY1 human αSyn-specific mouse monoclonal antibodies were used for ELISA (antibodies were developed in-house, commercially available as catalogue no. MABN1817 for 2F12, MABN1818 for SOY1, Sigma-Aldrich). Site-directed mutagenesis epitope mapping showed that 2F12 binds most strongly to a C-terminal epitope corresponding to amino acids 125–135, while SOY1 binds most strongly to a mid-region epitope corresponding to amino acids 91–110. For comparison, sections from the same blocks were stained with both 2F12 and LB509, a commonly used antibody in neuropathological diagnosis of synucleinopathies (Supplementary Fig. 2).

### Immunohistochemical staining and analysis of tissue

During frozen tissue dissection, a slice of ∼4-mm thick was taken from the area adjacent to the section analysed for αSyn content and stained as described (Sanderson, 2019). In short, the slice was submerged in 4% paraformaldehyde in phosphate-buffered saline (PBS) for between 72 and 96 h for fixation. Tissue was dehydrated and embedded in paraffin. Six-micrometre sections were cut and affixed to glass slides and dried overnight at 37°C. To remove the paraffin, slides were incubated for 3 min twice each in 100% ethanol, 95% ethanol and ultrapure water. Slides were then heated for 15 min by microwave in 10 mM sodium citrate and allowed to cool to ambient temperature before a 1-h ambient temperature incubation in 2% vol/vol fat-free milk in PBS-T. Staining was performed using 2F12 mouse monoclonal antibody to αSyn at a concentration of 65 ng/ml for 1 h at ambient temperature. Following incubation in primary antibody, slides were rinsed three times in PBS-T and then incubated for 2 h at ambient temperature in biotinylated anti-mouse IgG antibody (Southern Biotech, Birmingham, AL, USA) prepared at 2.5 μg/ml in 2% vol/vol fat-free milk in PBS-T. Following incubation, slides were rinsed twice in PBS-T and once in PBS, incubated in ABC avidin–biotin peroxidase complex (Vector Laboratories, Burlingame, CA, USA) for 1 h at ambient temperature, and then rinsed twice in PBS-T and once in PBS. Immunostaining was visualized using 3,3-diaminobenzidine horseradish peroxidase substrate (Vector Laboratories, Burlingame, CA, USA). Slides were rinsed three times in ultrapure water, counterstained with haematoxylin, differentiated in 0.3% acid alcohol and dehydrated by submerging in two baths of each of the following: 95% ethanol, 100% ethanol and 100% xylene. Glass coverslips were mounted in Permount (Thermo Fisher Scientific, Waltham, MA, USA).

### Denaturing α-synuclein sandwich enzyme-linked immunosorbent assay

All reagents were purchased from Meso Scale Discovery (Rockville, MD, USA). Prior to ELISA αSyn quantification, aliquots of all previously non-denatured samples were boiled for 10 min at 100°C in 2% SDS. Single-spot, standard-bind plates were coated with 200 ng of 2F12 prepared in PBS and incubated overnight at 4°C. Before sample loading, excess capture antibody was rinsed out and the wells were treated with 5% MSD Blocker A in TBS-T for 1 h at ambient temperature with shaking at 500 rpm. Blocking buffer was removed, and the plate was rinsed three times with TBS-T. Prior to loading, samples were diluted in 1% Blocker A with 0.5% Nonidet P-40 substitute in TBS-T to a final SDS concentration of no greater than 0.4%. Samples were loaded in duplicate, including amino acid sequenced recombinant αSyn standards diluted in the appropriate detergent mixture. Plates were shaken at 500 rpm for 1 h at ambient temperature and then incubated overnight at 4°C. Sample liquid was removed, the plate was rinsed three times with TBS-T and 200 ng of sulfo-labelled SOY1 diluted in 1% Blocker A was added per well. The plate was light-shielded and shaken for 1 h at ambient temperature at 500 × g. The detection antibody solution was then tapped out, and the plate was washed three times in TBS-T. About 150 μl of 2× Read Buffer S (diluted in ultrapure water) was added per well, and the plate was immediately read using a SECTOR Imager 2400.

### Size-exclusion chromatography fractionation of cytosolic proteins

Between 1 and 2.5 mg of total protein (measured via Pierce BCA kit; Thermo Scientific, Waltham, MA, USA) in 400–600 μl was injected into a 2--ml sample loop. The sample was passed over a Superose 6 Increase 10/300GL size-exclusion column (GE Healthcare, Pittsburgh, PA, USA) mounted on an ÄKTA chromatography system (GE Healthcare). The column was equilibrated with 50 mM ammonium acetate (pH 7.40). Using a flow rate of 1.5 ml/min, 1 ml of fractions were collected. Twenty microlitres from each fraction was mixed with 5 μl of 10% (v/v) SDS (final SDS concentration: 2%) and boiled for 10 min at 100°C for ELISA quantification. The remaining portion of the fractions were flash-frozen in liquid nitrogen and either stored at −80°C or lyophilized for future analysis. For the estimation of MW, a gel filtration molecular marker kit with standards ranging from 29 to 700 kDa in size (catalogue no. MWGF1000; Sigma-Aldrich) was used to calibrate the column prior to the analysis of brain material. These standards were used to generate a linear model relating elution volume to molecular radius.

### Permeabilization assays

The membrane permeabilization assay was performed as previously described (Flagmeier *et al.*, 2017). Briefly, 200 mM 1-palmitoyl-2-oleoyl-sn-glycero-3-phosphocholine (POPC) (16:0–18:1 PC: 18:1–12:0 Biotin PC—100:1) vesicles were prepared by extrusion and five freeze-thaw cycles. The lipid mixture was then hydrated in 100 µM Cal-520 dye dissolved in HEPES buffer (50 mM, pH 6.5). Vesicles were tethered to PLL-g-PEG coated cover slides via biotin–neutravidin linkage. The HEPES buffer was replaced with 50 µl Ca^2+^-containing buffer solution and blank images (*F*_blank_) were recorded. Each field was selected using a homemade stage-control programme to avoid user bias. Once these fields were selected, 50 µl of the soluble HMW fractions were added to the coverslips and incubated for 20 min before the same fields were re-imaged (*F*_sample_). Ionomycin was added as a positive control, and the same area containing vesicles are reimaged (*F*_ionomycin_). The relative influx of Ca^2+^ into an individual vesicle due to aggregates of αSyn was then determined using the following equation

$${\mathrm{Ca}}^{2+}\;\mathrm{influx}\;=\;\frac{F_{\mathrm{sample}}\;-\;F_{\mathrm{blank}}}{F_{\mathrm{ionomycin}}\;-\;F_{\mathrm{blank}}}.$$

For antibody experiments, the samples were incubated with 300 nM of antibody for 20 min before addition to the coverslips. Imaging were performed using a home-built total internal reflection fluorescence microscope equipped with 60×, 1.49 NA oil immersion objective lens. A 488-nm laser was used to excite the Cal-520 dye, and the emitted fluorescence was collected by electron multiplying charge-coupled device (CCD) camera.

### Cell lines

All materials purchased from Thermo Fisher Scientific unless otherwise noted. Human embryonic kidney cells (HEK-293; ATCC number CRL-1573) were cultured at 37°C and 5% CO_2_ in Dulbecco’s modified Eagle’s medium supplemented with 10% foetal bovine serum, 100 U/ml penicillin, 100 μg/ml streptomycin, 2 mM l-glutamine, 1 mM sodium pyruvate and 1× minimum essential medium (MEM) non-essential amino acids solution. Monoclonal stable cell lines were generated by transfecting with pcDNA4-A53T-huαSyn-yellow fluorescent protein using Lipofectamine 2000 according to the manufacturer’s instructions. The transfected cells were cultured under zeocin selection, and individual clones were selected. N27 rat dopaminergic neural cell line was purchased from EMD Millipore (catalogue no. SCC048, Billerica, MA, USA) and cultured at 37°C with 5% CO_2_ in the Roswell Park Memorial Institute (RPMI) 1640 medium supplemented with 10% foetal bovine serum, 100 U/ml penicillin, 100 μg/ml streptomycin and 2 mM l-glutamine. Monoclonal stable cell lines were generated by transfecting with pcDNA4-hu αSyn using Lipofectamine 2000 according to the manufacturer’s instructions. The transfected cells were cultured under zeocin selection, and individual clones were selected. The clone with the highest αSyn expression was selected for our cell viability analysis.

### Cell viability analysis

Cells were seeded at a density of 6500 cells per well on a black poly-d-lysine (PDL) coated 96-well plate with clear bottom (catalogue no. 354363; Corning, Corning, NY, USA). After 24 h of culturing, cells were treated with brain extract material for 72–96 h prior to viability measurement. Twenty-four hours prior to measurement, 1 μM staurosporine was added to three wells as a positive control for cytotoxicity. Two hours prior to measurement, culture medium was replaced with 120 μl of culture medium with 16% (v/v) CellTiter Blue reagent (Promega, Madison, WI, USA). Cells were cultured for 2 h, and then fluorescence was quantified using a BioTek H1 plate reader (BioTek, Winooski, VT, USA) with an excitation wavelength of 560 nm and an emission wavelength of 590 nm. The average fluorescence value of three non-cell containing wells was considered background and subtracted from all other values during analysis.

### A53T-α-synuclein-yellow fluorescent protein inclusion formation assay

This protocol has been adapted from Woerman *et al.* (2015). All reagents were purchased from Thermo Fisher Scientific unless otherwise noted. HEK293 cells stably expressing pcDNA4-A53T-hαSyn-yellow fluorescent protein were seeded at a density of 5000 cells per well on a black PDL-coated 96-well, clear-bottom plate (catalogue no. 354363; Corning). Protein samples (either recombinant monomer or pre-formed fibrils) were prepared in Opti-MEM with 3% (volume:volume) Lipofectamine 2000 and incubated at room temperature for 2 h. Prior to cell treatment, protein-Opti-MEM-Lipofectamine mixtures were mixed 1:1 with HEK culture medium. Culture medium on cells was removed and replaced by 100 μl per well of these proteofection mixtures. Cells were cultured for 4 h with the proteofection mixture, after which the mixture was removed and replaced with 250 μl of culture medium. The cells were cultured for 96 h, and fluorescent images were captured every 2 h using the IncuCyte Zoom live cell imaging platform (Essen Biosciences, Ann Arbor, MI, USA). Inclusion formation assessment was performed using the IncuCyte analysis software.

### Enrichment of Lewy body-associated brain material for bioactivity analysis

LB-rich sequential extractions were conducted according to the protocol published by Peng *et al.* (2018). Brain pieces of 200–400 mg were Dounce homogenized in four volumes (weight:volume) of high salt buffer with PIs (HS/PI) (50 mM Tris–HCl, 750 mM NaCl, 5 mM ethylenediaminetetraacetic acid (EDTA), pH 7.4 with complete PI tablet; Sigma-Aldrich). Homogenates were centrifuged at 100 000 × *g* for 30 min at 4°C. Supernatants were collected, and bicinchoninic acid assay for total protein concentration was performed to assess total protein concentration. Pellets were then re-extracted with HS/PI buffer, followed by sequential extractions with 1% Triton X-100-containing HS buffer, 1% Triton X-100-containing HS buffer with 30% (weight:volume) sucrose and finally 1% sodium lauroyl sarcosinate (sarkosyl) (weight:volume)-containing HS buffer. The HS + 1% sarkosyl homogenate was incubated with nutation overnight at 4°C. Following this incubation, the homogenate was centrifuged at 100 000 × *g* for 30 min and the supernatant was collected. The pellet was resuspended for washing in a volume of PBS equal to the homogenate volume initially centrifuged and then centrifuged at 100 000 × *g* for 30 min. The pellet was then resuspended and homogenized in 0.2 volumes of the initial homogenate volume of PBS using a probe sonicator. (catalogue no. 431MPX, QSonica, Newtown, CT, USA) for 30 s (5 s ON, 5 s OFF) at amplitude 20. After sonication, these samples were centrifuged at 100 000 × *g* for 20 min at 4°C. The supernatant was collected and filtered through a syringe filter (catalogue no. SLGV004SL; Millipore Sigma) in a cell culture hood. The sterile supernatant was then aliquoted in such that each aliquot contained 150 ng of total protein based on the bicinchoninic acid protein quantification. The aliquots were snap-frozen in liquid nitrogen in 0.5-ml Eppendorf Protein LoBind Tubes (catalogue no. 022431081, Eppendorf) and stored at −80°C.

### Harvest, culture and treatment of hippocampal primary rat neurons

The hippocampi of E18 SD rats were dissected in Hank’s Balanced Salt Solution buffered with HEPES and dissociated with 0.125% trypsin (Invitrogen, Carlsbad, CA, USA) for 15 min at 37°C followed by trituration. Dissociated cells were plated in 96-well plates pre-coated with poly-d-lysine (100 μg/ml) at a density of 15 000 cells/cm^2^. After culturing for 4 days in the Neurobasal medium with B-27 supplement and Glutamax (Invitrogen), FUDR (Sigma-Aldrich) was added to block astrocyte growth to a final concentration of 50 μg/ml. Half of the growth medium was exchanged every 4 days. Primary rat hippocampal neurons were cultured for 14 days *in vitro* (DIV14) as described previously (Jin *et al.*, 2011). At DIV14, pooled and lyophilized HMW SEC fractions (2–8) were resuspended in culture medium and applied to the cells in triplicate. Neurite length was monitored over 72 h using the IncuCyte live cell imaging platform (Essen Biosciences).

### Culture and treatment of human induced pluripotent stem cell-derived neurons

The induced pluripotent stem cell (iPSC) line used in this study (YZ1) was originally generated from the IMR-90 cell line (ATCC) and characterized as described previously (Zeng *et al.*, 2010). Due to a karyotype abnormality in a small subset of cells, monoclonal isolates were obtained and confirmed to be karyotypically normal and pathogen-free prior to this study (Srikanth *et al*., 2018). The iPSC line used was confirmed to be of the correct identity using short tandem repeat profiling (Genetica Cell Line Testing). iPSCs were maintained in media containing 400 ml of Dulbecco’s modified Eagle’s medium/F12 (Invitrogen), 100 ml of KnockOut Serum Replacement, 5 ml of penicillin/streptomycin/glutamine, 5 ml of MEM non-essential amino acids and 500 μl of 2-mercaptoethanol (all from Invitrogen) with fresh addition of 10 μg/ml fibroblast growth factor-basic (bFGF) (Millipore). The differentiation was achieved through neurogenin expression under a doxycycline promotor as previously reported with minor modifications (Zhang *et al.*, 2013). Induced neurons, which are described as a homogenous population of neurons expressing VGLUT2, a transporter for the excitatory neurotransmitter glutamate, were plated at DIV4 on 96-well plates (Greiner Bio-One, Monroe, NC, USA) pre-coated with Matrigel (Corning) at a density of 5000 cells per well and maintained in media consisting of 1% Glutamax (volume:volume), 0.3% dextrose (weight:volume) and 0.5% MEM non-essential amino acids (volume:volume) in the Neurobasal medium. Two percent B27 supplement (v/v), brain-derived neurotrophic factor (BDNF), ciliary neurotrophic factor (CNTF), glial cell line-derived neurotrophic factor (GDNF) (10 ng/ml each), puromycin (1 μg/ml) and doxycycline (2 μg/ml) were added just prior to feeding. Differentiated cells were treated between DIV21 and DIV28 with insoluble extracts prepared as described above containing 50 ng of total protein per well and brought to a final volume of 200 μl. Cells were treated in triplicate wells with randomized locations. During the course of the treatment, the cells underwent a single medium replacement of 50% of the volume in each well.

### Neurite morphology analysis

The IncuCyte automated live cell imaging platform captured phase contrast images every 2 h over a total of 88–96 h. Neurite and cell body masks were applied to the images (Supplementary Fig. 3). Average neurite length and cell body cluster area were measured using the IncuCyte image capture and analysis software in Neurotrack mode. To compare neurite degeneration between wells, average neurite lengths at 88 h were divided by the normalized average neurite lengths in the same field at 0 h. This was compared to the same ratio in untreated wells.

### Statistical analysis

Prism (GraphPad, San Diego, CA, USA) was used for all statistical analyses and graph generation. Details of each analysis are provided in the relevant figure legends. Means ± standard error of the means (SEMs) are shown in cohort-wide analyses, while means ± standard deviations are shown in single-patient analyses.

### Data availability

The data that support the findings of this study are available from the corresponding author upon request.

## Results

### α-Synuclein is differentially distributed in subcellular compartments in dementia with Lewy Bodies cortical tissue

**Figure 1:**
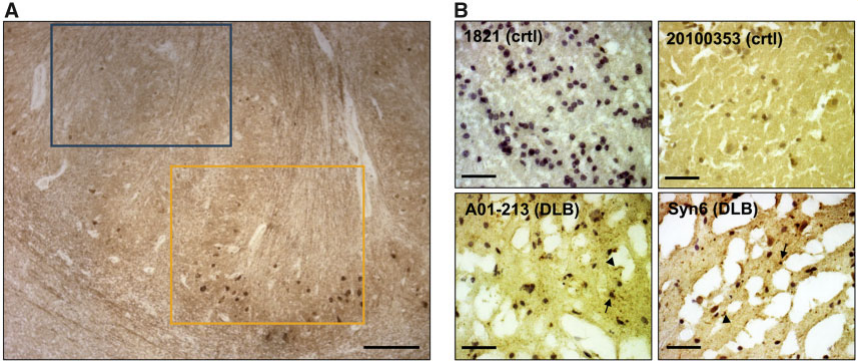
**IHC confirmation of synucleinopathy in DLB frontal cortical samples.** (**A**) IHC of the formalin-fixed striatum of MGH DLB brain #1594 with αSyn monoclonal antibody 2F12 demonstrates the striking local heterogeneity of cytopathology. Yellow box: area of high density of Lewy cytopathology; blue box: immediately adjacent area devoid of IHC-detectable αSyn aggregates. Scale bars: 300 μm. (**B**) Small frozen pieces of tissue were excised from locations adjacent (within ∼5 mm) to the frozen pieces we used for our biochemical extractions. The resultant cryostat sections were stained with αSyn monoclonal antibody 2F12 and nuclei counterstained with haematoxylin. Representative images from two control and two DLB brains show ice crystals resulting from variable freezing of the unfixed tissue. Arrows exemplify LBs; arrowheads exemplify Lewy neurites. Scale bars: 80 μm.

**Figure 2:**
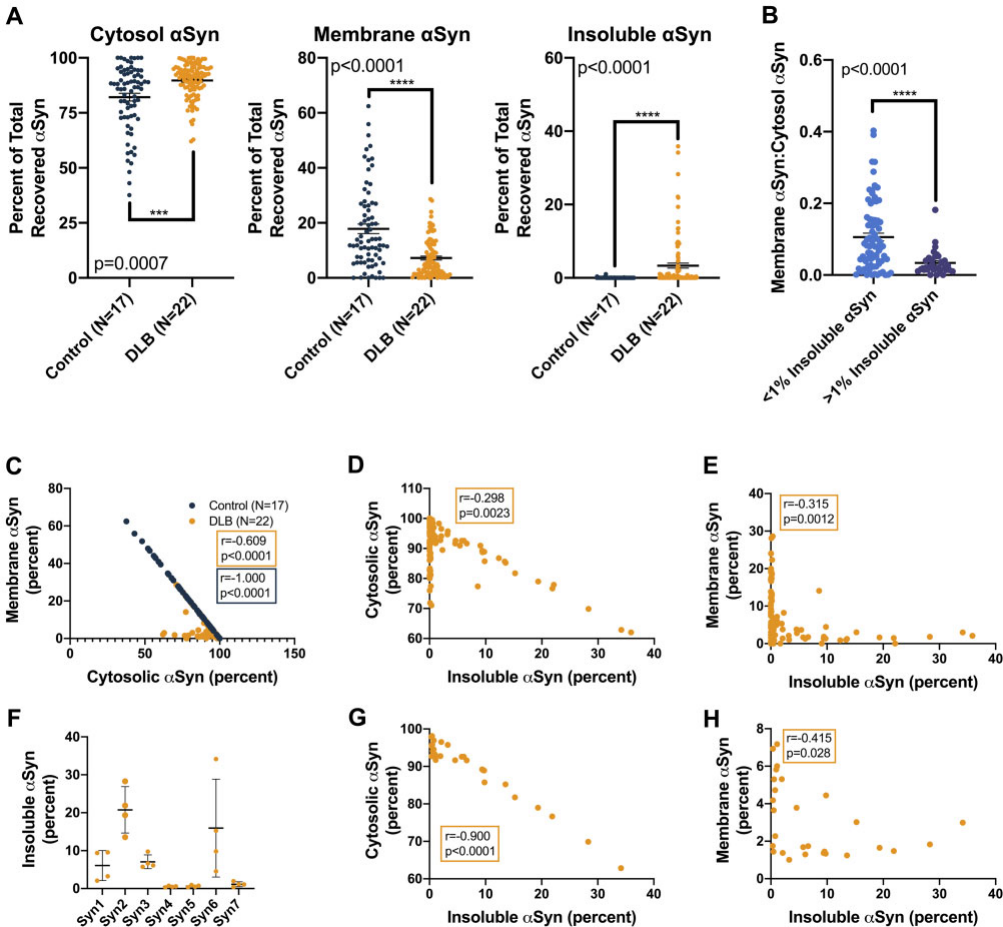
**αSyn solubility distribution in DLB versus control brains based on the percentage of total analysis.** (**A**) For each tissue piece (represented by a single point), the ELISA-measured αSyn concentration was multiplied by the original volume of the extract, yielding the total αSyn in each fraction. This was then expressed as a percentage of the total αSyn recovered in all three fractions. Graphs: percentage of total αSyn in the cytosolic, membrane (1% Triton-soluble) and insoluble (SDS/8 M urea-soluble) extracts. Between three and eight tissue pieces were processed and analysed per brain, and each piece was measured in triplicate. Bars: means with SEMs. Control and DLB extracts were compared using the Mann–Whitney comparison of ranks. (**B**) DLB brain extracts were stratified based on insoluble αSyn quantities (blue: <1% of total extracted αSyn; purple: >1% of total extracted αSyn), and the ratio of membranous to cytosolic αSyn was calculated for each group. Proportionally, this analysis revealed higher membranous αSyn in low-pathology DLB extracts (*P* < 0.0001, Mann–Whitney comparison of ranks). (**C**) Correlation analyses of the percentages of recovered αSyn in the cytosolic and membrane cortical fractions, both from control subject and DLB patient brains, showed strong negative associations, an unsurprising finding given that these fractions contain most of the total extracted αSyn (control: Spearman *r* = −1.000, 95% CI: −1.000 to −1.000, 2-tailed *P* < 0.0001; DLB: Spearman *r* = −0.609, 95% CI: −0.721 to −0.462, *P* < 0.0001). (**D** and **E**) Strong negative associations also exist between the percentage of total extracted αSyn in the insoluble fraction and the percentage of total extracted αSyn in both the cytosolic and membrane-associated fractions across all cortical extracts from DLB patient brains (insoluble versus cytosol: Spearman *r* = −0.298, 95% CI: −0.469 to −0.105, *P* = 0.0023; insoluble versus membrane: Spearman *r* = −0.315, 95% CI: −0.484 to −0.124, *P* = 0.0012). (**F**) In terms of insoluble αSyn as a percentage of total extracted αSyn, DLB patient brains from the Mayo Clinic brain bank showed the highest pathological burden. Means with standard deviations are shown. (**G** and **H**) Correlation analyses comparing the percentage of total extracted αSyn in insoluble fractions to cytosolic and membrane-associated fractions from these high-burden Mayo Clinic brains showed even stronger negative associations than the analyses that included DLB patient brains from all sources (insoluble versus cytosol: Spearman *r* = −0.900, 95% CI: −0.954 to −0.89, *P* < 0.0001; insoluble versus membrane: Spearman *r* = −0.415, 95% CI: −0.689 to −0.386, *P* = 0.0028).

Using sequential extraction with solvents of increasing denaturing capability, we quantitatively analysed the relative solubility of αSyn in frontal cortical extracts from 22 DLB patients and 18 non-synucleinopathy control subjects (Table 1). Analysis of demographic data showed no difference in ages or post-mortem intervals between the two populations (*P* = 0.929 and *P* = 0.980, respectively, unpaired *T*-tests). No post-mortem interval data were provided for 6 of the 22 DLB brains and 4 of the 18 control brains, and no ages were given for one of the 18 control brains. In addition, chi-square analysis showed no significant deviation from the expected gender ratios (*P* = 0.262) (Table 2). To minimize the significant variation we observed in both the levels of ‘insoluble’ αSyn (insoluble in OG-RIPA but soluble in 5% SDS/8 M urea-soluble) and the degree of Lewy cytopathology by αSyn immunohistochemistry (IHC) (Fig. 1), we homogenized between three and six different tissue pieces dissected from frozen frontal cortex of each DLB brain that showed Lewy pathology by αSyn IHC in sections prepared from a piece of the adjacent frozen cortex. When thawed, each of these pieces weighed ∼50 mg. Small aliquots of each extraction fraction were denatured by boiling in 2% SDS to measure total αSyn amount by sandwich ELISA. To account for possible shifts in cell populations in diseased tissue, such as the presumed loss of αSyn-expressing neurons and the local invasion of non-expressing glia, we expressed the data in αSyn levels as a percentage of the total αSyn recovered from all three fractions (Fig. 2). We also expressed the absolute amount of αSyn in each sequential extract normalized to total protein (per a bicinchoninic acid total protein assay) in Supplementary Fig. 4.

**Table 2:** Unpaired *T*-tests showed no significant difference between the mean ages or PMIs of the control and DLB patient samples

	Control (*N* = 18)	DLB (*N* = 22)	*P*-value
Age	72.8 ± 13.5	72.5 ± 8.6	0.93
PMI	24.1 ± 20.9	23.9 ± 20.5	0.98
Gender (F:M)	11:7	9:13	0.26

A chi-square test also showed no significant difference in the F:M reported gender ratios between the groups.

F: female; M: male; PMI: post-mortem interval.

While this extraction was designed primarily to identify small brain pieces with high pathology burden based on the established finding that detergent-insoluble αSyn is associated with high histopathological LB density (Campbell *et al.*, 2000; Klucken *et al.*, 2006), we also included a membrane-associated fraction to examine the subcellular localization of αSyn in our relatively large, multi-centre collection of human samples. Expressing the three extract fractions (cytosol, membrane extract and urea-solubilized extract) as a percentage of total recovered αSyn showed differences in the solubility distributions of αSyn extracts from control compared with synucleinopathy tissue. DLB cytosols contained a higher proportion of extracted αSyn than those of controls, whereas membrane-associated fractions showed the opposite: DLB samples had a lower proportion of total extracted αSyn (Fig. 2A). This observation suggests a partial redistribution of membrane-bound αSyn to the cytosolic compartment in the affected DLB cortex. Although correlation analyses of related percentages, by definition, show strong associations, we interrogated whether the relative strengths of these associations on a sample-to-sample basis suggested a predictable redistribution of αSyn from one compartment to another. Stratification of DLB brain extracts into low αSyn burden (insoluble αSyn < 1%) and high αSyn burden (insoluble αSyn > 1%), and analysis of membrane:cytosol αSyn ratio showed a relative shift to the cytosolic compartment in high-insoluble αSyn brains, suggesting that insoluble αSyn may come from the membrane compartment (Fig. 2B). This result supports recent research showing an abundance of vesicle-bound αSyn in Lewy bodies (Shahmoradian *et al.*, 2019) and reinforces the hypothesis that vesicle binding increases αSyn aggregation (Galvagnion *et al.*, 2015). As expected, in control subject brain samples, there was nearly perfect linear correlation between the cytosolic and membrane-associated αSyn, as physiological αSyn likely exists in a rapid equilibrium between its soluble and membrane-bound forms [Spearman *r* = −1.000, 95% confidence interval (CI): −1.000 to −1.000, *P* < 0.0001] (Fig. 2C). Because the αSyn extracted from DLB patient brains was spread across all three fractions, with variable elevations in the final urea-solubilized extract (Fig. 2A, right), this correlation was not as strong in DLB (Spearman *r* = −0.609, 95% CI: −0.721 to −0.462, *P* < 0.0001) (Fig. 2C). Looking exclusively at DLB patient brains, there were strong negative associations between the insoluble fractions and the cytosolic and membrane fractions (insoluble versus cytosol: Spearman *r* = −0.298, 95% CI: −0.469 to −0.105, *P* = 0.0023; insoluble versus membrane: Spearman *r* = −0.315, 95% CI: −0.484 to −0.124, *P* = 0.0012) (Fig. 2D and E). In analysis of the brains obtained from the Mayo Clinic brain bank, which had exceptionally high levels of insoluble αSyn (Fig. 2F), both of these negative correlations were even stronger. This suggests that in patients with a high burden of LB pathology, αSyn from both the cytosolic and membrane-associated physiological pools contributes to the formation of the insoluble aggregates (insoluble versus cytosol: Spearman *r* = −0.900, 95% CI: −0.954 to −0.89, *P* < 0.0001; insoluble versus membrane: Spearman *r* = −0.415, 95% CI: −0.689 to −0.386, *P* = 0.0028) (Fig. 2G and H).

Looking at the absolute αSyn concentrations in each fraction, we observed significantly lower levels of membrane-associated αSyn in DLB than control cortex when normalized to total protein (*P* < 0.0001) (Supplementary Fig. 4A) and robust regression and outlier removal outlier removal did not alter this finding. αSyn content in the urea-soluble extracts confirmed that DLB cortex had greater levels of highly insoluble αSyn than did control cortex (*P* < 0.0001) (Supplementary Fig. 4A). As expected, only negligible amounts of αSyn were detected in the urea-solubilized fraction of control brains, indicating that the αSyn detected in this fraction derives from the hallmark aggregated αSyn lesions of DLB.

Analyses of the of log-normalized absolute αSyn levels (Supplementary Fig. 4B–D) in the three different extracts revealed associations of αSyn levels between fractions. Cytosolic absolute αSyn levels from frontal cortex (whether DLB or control) correlated strongly with membrane-associated absolute αSyn levels (control: Spearman *r* = 0.656, *P* < 0.0001, 95% CI: 0.494–0.770, DLB: Spearman *r* = 0.761, *P* < 0.0001, 95% CI: 0.662–0.834) (Supplementary Fig. 3B). Detergent-insoluble/urea-solubilized (Lewy-associated) αSyn levels showed no significant relationships with membrane or cytosolic αSyn levels in DLB extracts (insoluble versus cytosol: Spearman *r* = −0.078, 95% CI: −0.118 to −0.267, *P* = 0.436; insoluble versus membrane: Spearman *r* = −0.026, 95% CI: −0.174 to −0.224, *P* = 0.793) (Supplementary Fig. 4C and D), except for a subset of DLB samples with the most severe LB/Lewy neurite pathology burden by high levels of insoluble αSyn in the corresponding urea extracts. Specifically, DLB frontal cortices from the Mayo Clinic brain bank that had very high levels of insoluble, Lewy-associated αSyn (Supplementary Fig. 4E–G), showed strong negative correlations between those levels and both cytosolic and membrane-associated αSyn (insoluble versus cytosol: Spearman *r* = −0.719, 95% CI: −0.864 to −0.464, *P* < 0.0001; insoluble versus membrane: Spearman *r* = −0.563, 95% CI: −0.778 to −0.230, *P* = 0.0018).

Analysis of this protein-normalized αSyn content in each fraction revealed a significant decrease in the mean level of total extracted αSyn in DLB frontal homogenates versus controls by ∼25% (*P* = 0.0016, Mann–Whitney comparison of ranks) (Supplementary Fig. 4A). Analysis of cytosolic fractions yielded a similar result: on average, there was significantly less αSyn in DLB extracts than in control (*P* = 0.010) (Supplementary Fig. 4A).

Notably, analysis comparing the subcellular localization of αSyn in extracts of two brains of patients with familial DLB (one A53T αSyn mutant and one *SNCA* duplication carrier) did not show striking differences compared with the pooled data from all control extracts (Supplementary Fig. 5A). Statistical analyses were not performed due to the disparate sample sizes.

### Soluble, high molecular weight α-synuclein species are more abundant in dementia with Lewy Bodies than control cortical extracts

**Figure 3:**
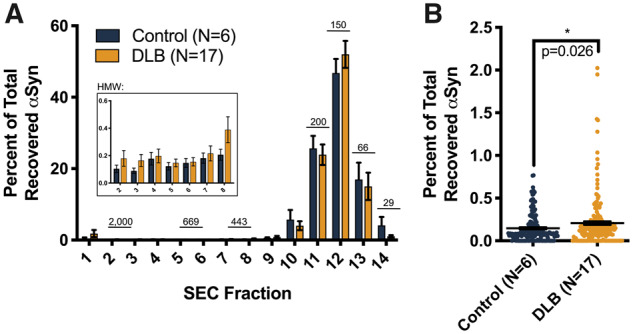
**Size distribution of soluble αSyn from DLB and control frontal cortex.** TBS-soluble, post-175 000 × *g* supernatants from cortical pieces with high levels of insoluble (Lewy-associated) αSyn and corresponding controls were fractionated by SEC (3 MDa exclusion size; see Materials and methods). To correct for the amount of αSyn injected into the SEC column for each case, αSyn levels for each fraction were expressed as a percentage of total αSyn recovered across all the SEC fractions. (**A**) Percentage of total αSyn in each SEC fraction (means with SEMs are shown); elution of marker proteins indicated in kDa. Inset: αSyn recovered in HMW fractions 2–8, corresponding to MWs from ∼2 MDa to ∼440 kDa. (**B**) Pooling the HMW fractions revealed significantly higher mean αSyn levels in DLB cytosolic extracts than controls (*P* = 0.0256, Mann–Whitney comparison of ranks). DLB: *n* = 1–4 cytosolic fractions per subject; controls: *n* = 1–8.

The cytosolic fractions of brain pieces with high levels of αSyn in the urea extracts were fractionated using non-denaturing SEC on a column with a 3-MDa exclusion size to probe for relative abundance of a range of HMW forms of soluble αSyn. After SEC fractionation, small aliquots of each fraction were boiled in 2% SDS for 10 min to denature the samples and maximally expose epitopes prior to αSyn ELISA quantification. Across all samples, an average of 91.2% of soluble αSyn eluted in SEC fractions 11–13, which correspond to the expected elution volume of physiological αSyn (MW of ∼80 kDa based on the elution of recombinant protein standards; Supplementary Fig. 6). Comparison of αSyn levels in individual SEC fractions of control and DLB cytosols revealed no statistically significant differences (Fig. 3A). However, pooling the individual levels across the HMW SEC fractions (i.e. fractions 2–8, corresponding to ∼2 MDa down to ∼440 kDa, Supplementary Fig. 6) revealed a significant increase in relative levels of soluble, HMW αSyn in cytosols of DLB versus control cortex (*P* = 0.0256, Mann–Whitney comparison of ranks) (Fig. 3B). On average, the αSyn content of these HMW SEC fractions comprised only 1.0% (in control cortex) and 1.4% (in DLB cortex) of the total eluted cytosolic αSyn, indicating that in the diseased brain, HMW-soluble αSyn species is relatively elevated but still not abundant, similar to previous observations of tau protein species present in Alzheimer’s disease brain (Takeda *et al*., 2015).

### Soluble α-synuclein species increase the permeability of lipid membranes *in vitro*

**Figure 4:**
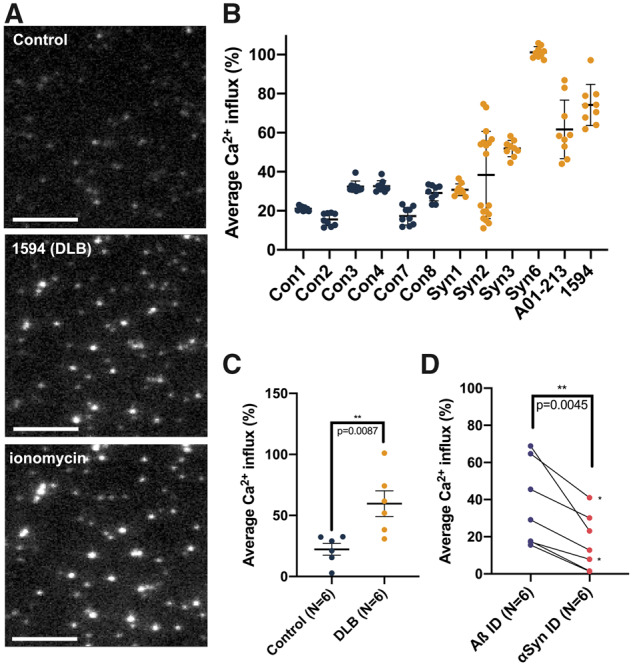
**Quantitative measurement of membrane permeabilization upon applying HMW fractions of soluble brain extracts via fluorescence imaging.** (**A**) Images obtained through TIRF microscopy show an untreated vesicle sample (*top*, ‘Control’), a vesicle sample treated with soluble, HMW brain material from DLB patient 1594 (*middle*) and a sample treated with ionomycin as a positive control (*bottom*). Scale bars are 3 μm. (**B**) Brain homogenates display variable vesicle permeabilization capability between different cases but low variability within replicates of the same samples. Means with standard deviations are shown. (**C**) Mann–Whitney comparison of ranks analysing average Ca^2+^ influx measured from six DLB and six control brains reveals significantly elevated permeabilization of lipid membranes by soluble extracts from DLB patients (*P* = 0.0087). For each patient sample, nine technical replicates were used to generate the data point. Means with SEMs are shown. (**D**) Immunoneutralization of DLB cytosolic extracts with 2F12 anti-αSyn antibody (2F12, right) reduces the DLB extract-induced permeabilization of the vesicles compared to immunoneutralization anti-Aβ antibody (4G8, left) (paired *T*-test, *P* = 0.0045). For each patient sample, nine technical replicates were used to generate the data point. Each pair of points connected by a line represents a single cytosolic HMW extract. The two pairs of points denoted by a ‘*’ came from a single DLB brain (Syn2).

We then tested whether the soluble, HMW SEC fractions of brain cytosols were capable of phospholipid membrane permeabilization. We used a previously described membrane permeabilization assay that utilizes nanosized lipid vesicles tethered to a coated glass surface via biotin–neutravidin linkage and filled with a Ca^2+^-sensitive dye (Flagmeier *et al.*, 2017). Influx of Ca^2+^ into these vesicles from the surrounding buffer was measured using total internal reflection fluorescence microscopy (Fig. 4A). We applied the pooled HMW SEC fractions (fractions 2–8, corresponding to a molecular weight of 2 MDa–440 kDa) from cytosolic extracts of six DLB and six control brains to this assay. Extracts from both subject groups caused influx of Ca^2+^, indicating the destabilization of lipid membranes (Fig. 4B). On average, application of pooled HMW fractions cytosolic extracts from DLB brains destabilized membranes significantly more than corresponding extracts from control subject brains (Mann–Whitney comparison of ranks, *P* = 0.0087; Fig. 4C). Interestingly, samples from brains Syn3 and Syn6 caused complete permeabilization of lipid vesicles. Importantly, immunoneutralization of these samples with an αSyn-specific monoclonal antibody (2F12), but not a beta-amyloid (Aβ)-specific monoclonal antibody (4G8), markedly decreased the Ca^2+^ influx (paired *T*-test, two-tailed *P* = 0.0045). This indicates that the active species in these DLB cortical fractions that potently enhance membrane permeabilization are composed of αSyn (Fig. 4D). This finding provides validating human support of similar results obtained in both *in vitro* and mouse models with αSyn fibrils (Fusco *et al.*, 2016; Mor *et al.*, 2017). Interestingly, despite unremarkable shifts in subcellular localization of αSyn compared with control extracts (Supplementary Fig. 5A), the two familial DLB brain extracts significantly increased the permeabilization of these vesicles (Supplementary Fig. 5B, Mann–Whitney comparison of ranks, *P* < 0.0001 for both brains). This increase in permeabilization appeared dependent on the presence of exposed αSyn epitopes (Supplementary Fig. 5C), although this dependence did not meet the level of statistical significance (paired *T*-test, *P* = 0.206).

### Insoluble α-synuclein from dementia with Lewy Bodies brain extracts induces morphological changes in human induced pluripotent stem cell neurons

**Figure 5:**
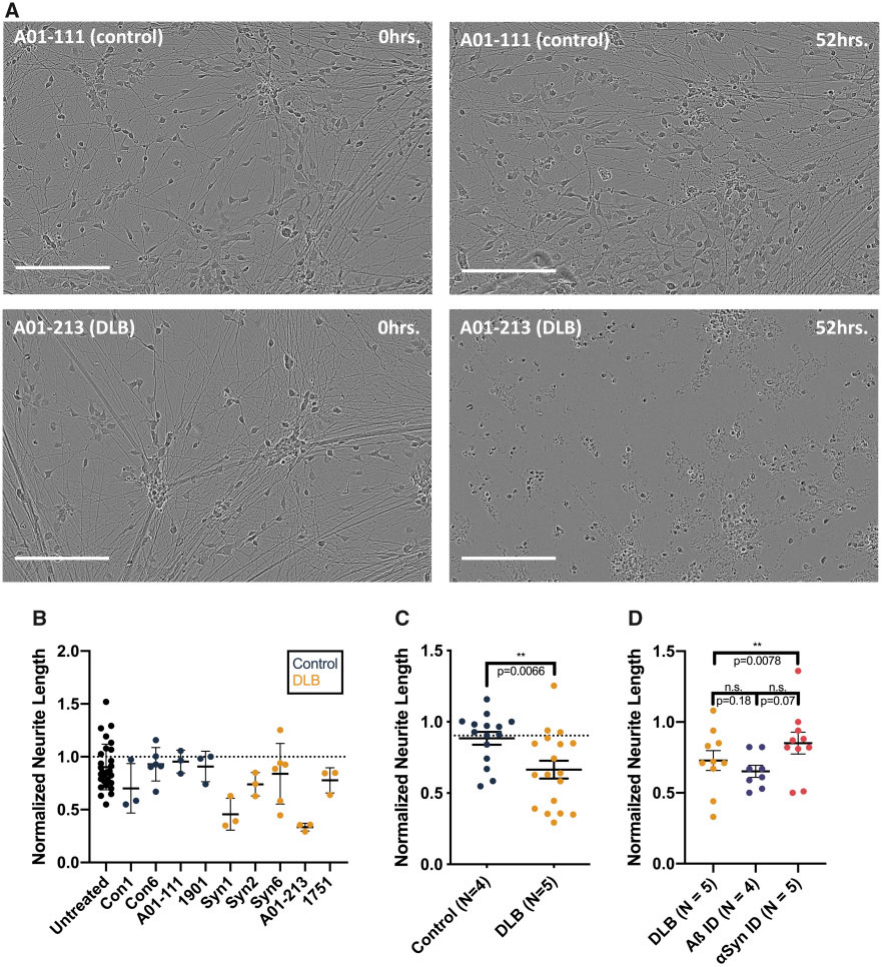
**Treating iPSC-derived human neurons with Lewy-associated brain extracts.** (**A**) Neurogenin-induced human neurons were treated with protein-normalized volumes of sonicated Lewy-associated brain extracts. Neurite morphology was monitored for 88–96 h. Images were analysed by applying a neurite mask (Supplementary Fig. 5) and automatically quantifying mean neurite length. In these representative images, neurons treated with αSyn-enriched control brain extract (*top*) show largely intact neurites at 52 h, whereas neurons treated with A01-213 DLB αSyn-enriched extract display neurite retraction (*bottom*). Images shown from each brain sample are taken from identical fields at different time points. Bars are 200 μm. (**B**) Mean neurite length calculated across four images per well at 88 h was normalized to the mean neurite length at *t* = 0. While the amount of αSyn levels applied to each well was not normalized, these levels did not correlate with the extent of neurite retraction (not shown). Means with standard deviations are shown. (**C**) Pooling of data collected from four non-synucleinopathy control extracts and five DLB patient extracts across three separate experiments shows a significantly greater neurite retraction in neurons treated with DLB extracts (*P* = 0.0066, Mann–Whitney comparison of ranks). Means with SEMs are shown. (**D**) Human iPSCs were treated with two extracts from five DLB brains, each split into three aliquots: one was applied directly; one was pre-incubated with 2F12, a monoclonal antibody to αSyn; and a third was pre-incubated with 4G8, a monoclonal antibody to Aβ. Immunoneutralization of αSyn in these extracts restored the length of neurons treated compared with neurons treated with non-immunoneutralized aliquots of the same DLB extracts (*P* = 0.0078, Wilcoxon paired comparison of ranks). Each point represents the mean of technical triplicates from a single experiment. Means with SEMs are shown.

To assess the potential neurotoxicity of insoluble, Lewy-associated αSyn, we generated a sonically dispersed preparation of detergent-insoluble cortical protein using density gradient centrifugation and partial detergent treatment (Blumenstock *et al.*, 2017). These sonicated brain extracts were added to a homogenous population of iPSC-derived, neurogenin-2-induced human excitatory neurons (Zhang *et al.*, 2013), and neurite morphology was monitored over 88 h (Fig. 5A and Supplementary Fig. 3). Previous works have shown that alterations in the morphology of neurite processes are sensitive indicators of neurotoxicity (Stephens *et al.*, 2005; Scott *et al.*, 2010; Domínguez-Álvaro *et al.*, 2018). Significantly, the particular commercial live-imaging platform used to assess morphological changes in neurons relies not on the expression of non-physiological, but rather on the light microscopy coupled with a computer algorithm for detecting and applying masks to either neurites or cell bodies. For this assay, we applied extracts from five DLB brains with the highest levels of detergent-insoluble/8 M urea-solubilized αSyn and four corresponding control brains. The effects of these extracts on neurite length showed significant variability across both control and DLB brain samples (Fig. 5B), but when data were aggregated based on neuropathological diagnosis, the DLB extracts led to significantly lower mean neurite length than did the control extracts (*P* = 0.0066, Mann–Whitney comparison of ranks) (Fig. 5C). Comparison of cells treated with Aβ-immunoneutralized extracts (4G8 antibody) with those treated with αSyn-immunoneutralized extracts (2F12 antibody) did not show a significant difference. However, there was a trend towards shorter neurite length in the Aβ-immunoneutralized extracts (*P* = 0.0703, Wilcoxon paired comparison of ranks), suggesting that the toxicity of these extracts is driven primarily by pathological αSyn, not Aβ (Fig. 5D).

## Discussion

Using non-denaturing biochemical fractionation of human frontal cortex to avoid potential denaturation of endogenous αSyn species (Luth *et al.*, 2015), we have characterized the oligomeric landscape of a variety of DLB and control brain tissue samples and validated the existence of neurotoxic soluble αSyn species in human brain. We report and describe a number of biochemical findings, tightly correlating with neuropathology, which could help elucidate the role of αSyn in the inception and progression of synucleinopathies. In addition, we identified pieces of tissue from our collection with both biochemical and neuropathological evidence of significant αSyn disease burden, establishing a method to identify pathology-rich tissue pieces for further analysis of corresponding soluble species. Notably, we observed a high degree of ‘microheterogeneity’ within single sections of tissue, both by IHC (Fig. 1) and biochemistry (Fig. 2). This observation drove our decision to look at several small tissue pieces per brain, as we feared that unburdened areas of larger sections of tissue might dilute any pathology-specific effects and we recommend future studies to employ a similar approach.

Through the quantification of αSyn levels in fractions generated through our extraction process, we observed differences in the subcellular distribution of αSyn species in DLB brains compared with controls. Analysis of our most pathology-rich tissue indicated that the αSyn levels in the cytosolic and membrane fractions of a single tissue piece were associated with the degree of Lewy neuropathology as determined by its level of insoluble αSyn. The strong inverse relationship between the relative levels of pathological, Lewy-associated αSyn and cytosolic or membrane-associated αSyn implies that the accumulation of insoluble αSyn is accompanied by a relative decrease in its physiological pools. Our observation that strong αSyn pathology is directly associated with a decrease in physiological αSyn in the same small samples of DLB cortex supports the possibility of a loss-of-function of physiological αSyn in the disease (Gorbatyuk *et al.*, 2010*a*, *b*; Benskey *et al.*, 2016).

Our analysis of the absolute concentrations of αSyn in different fractions points towards this possibility: in patients with DLB, there were lower total levels of αSyn, an expected consequence of late-stage disease with accompanying neuronal loss. At the same time, according to our analyses, the remaining αSyn redistributes to the cytosolic and urea-solubilized fractions. We found the relative biochemical increase in urea-solubilized αSyn to be associated with the presence of insoluble αSyn aggregates, which are the histopathological hallmarks of DLB and the other synucleinopathies. However, the relative increase in cytosolic αSyn in DLB cortex is not as readily explained. Our native SEC-ELISA analyses do show a significant increase in soluble, HMW species that could contribute to this redistribution; however, these species are of very low abundance, comprising ∼0.1–1% of the total extractable αSyn in our assays. Alternatively, the shift in relative abundance away from the membrane-associated fraction to the cytosolic fraction may be related to a loss of physiological function. Previous work has shown that transient interactions with phospholipid membranes induce proper folding and assembly of aggregation-resistant multimeric αSyn (Rovere *et al.*, 2018), and it appears that αSyn is likely involved in the physiological recycling of exocytotic (including synaptic) vesicles (Logan *et al.*, 2017). In addition, recent studies have shown that altering the lipid composition of vesicles can mitigate αSyn-related toxicity (Fanning *et al.*, 2019; Vincent *et al.*, 2018). Thus, it is possible that in the pathological state, more αSyn is in a disordered, cytosolic state that is more prone to aggregation than the multimeric state (Bartels *et al.*, 2011).

In further analysis of the cytosolic fractions, SEC-ELISA fractionation and analysis showed that, proportionally, HMW αSyn species are more abundant than in simultaneously and identically prepared extracts of age-matched control cortex. These soluble HMW species span a broad distribution of apparent sizes (440 kDa–3 MDa). Despite this finding, the low absolute levels of soluble HMW αSyn in both DLB and control cortex posed a challenge for further analyses and characterizations. In fact, the total amount of αSyn in these fractions, even when pooled, was close to the lower limit of quantification of our ELISA (0.5–1 ng/ml) than to the minimum concentration of synthetic αSyn pre-formed fibrils required to see an effect in our assays (500 ng/ml). While we observed no frank neurotoxicity of these fractions in a neurite outgrowth assay, our vesicle permeabilization assay indicated that these fractions can decrease phospholipid membrane integrity in an αSyn-dependent manner, an effect that likely contributes to neurotoxicity over time. The disparate sensitivities of our assays and the low concentrations of αSyn in these soluble extracts might explain the apparent lack of neurotoxicity. However, our findings in a vesicle permeabilization assay indicate that low amounts of HMW αSyn present in DLB brain cortex are qualitatively different than HMW αSyn present in the control cortex of aged brains and suggest a possible mechanism of toxicity.

We also examined the toxicity of the Lewy-associated αSyn species that are enriched in our detergent-insoluble, urea-solubilized fraction. These insoluble species alone demonstrated the ability to alter neurite morphology in human iPSC-derived neuronal cultures (Fig. 5), providing evidence that highly aggregated αSyn species found in LBs and Lewy neurites may contribute to the cytotoxicity that leads to the neurodegeneration and clinical manifestation of synucleinopathies. Given that the levels of cytosolic αSyn (including the soluble HMW αSyn species) correlate inversely with the levels of insoluble αSyn in our collection of DLB frontal cortices (Fig. 2F), we speculate that the soluble aggregates are an intermediate species that both directly contribute to neurotoxicity and deplete pools of physiological αSyn, analogous to the proposed role of amyloid plaques in Alzheimer’s disease (Martins *et al.*, 2008; Shankar *et al.*, 2008).

In conclusion, this study characterizes αSyn-specific biochemical differences in a large sample of DLB and control brain tissue for the first time. We also report on challenges associated with working with multiple brain banks. We hope that reporting on these challenges may standardize future tissue collection and classification protocols. In addition, bioassays of αSyn-enriched DLB samples provide more clues that should guide future research into the toxic mechanism of pathological αSyn.

## Supplementary Material

fcaa010_Supplementary_Data

## Abbreviations

αSyn =: α-synuclein
Aβ =: beta-amyloid
CI =: confidence interval
DLB =: dementia with Lewy Bodies
ELISA =: enzyme-linked immunosorbent assay
HMW =: high molecular weight
HS =: high salt buffer
IHC =: immunohistochemistry
iPSC =: induced pluripotent stem cell
LB =: Lewy body
OG-RIPA =: radioimmunoprecipitation assay
PBS =: phosphate-buffered saline
PD =: Parkinson’s disease
PI =: protease inhibitor
SDS =: sodium dodecyl sulphate
SEC =: size-exclusion chromatography
SEM =: standard error of the mean
