# Supplementary material for: Analysis of α-synuclein species enriched from cerebral cortex of humans with sporadic dementia with Lewy bodies
Source: Brain Commun. 2020 Feb 11;2(1):fcaa010. doi: 10.1093/braincomms/fcaa010 (PMC7130446; doi:10.1093/braincomms/fcaa010)
Supplement: fcaa010_Supplementary_Data [file fcaa010_supplementary_data.pdf]

SUPPLEMENTARY MATERIAL

Results

HMW soluble αSyn species show limited bioactivity in cell reporter assays

To search for possible effects of soluble αSyn on neuronal morphology and viability, primary rat hippocampal neurons were exposed to the pooled HMW SEC fractions, as described in the last section. Neurons were monitored for morphological change by automated light microscopy (IncuCyte; Essen) that quantified mean neurite length and branch point number over the course of a 72-hour exposure (**Supplementary Figure 7A**). Neither control nor DLB HMW SEC fractions altered neurite length or branch point number. The amount of HMW αSyn applied to these neurons was low (1-10ng/mL), and thus may be unlikely to trigger morphologic changes over the course of such a short exposure.

Next, in an HEK293 cell line stably expressing YFP-tagged human αSyn with the A53T fPD mutation, we tested the ability of both the HMW SEC-fractionated cytosol and resuspended insoluble extracts to trigger αSyn positive cytoplasmic inclusion formation. Protein from these respective brain fractions was introduced into the cells by Lipofectamine 2000 transfection (Woerman *et al.*, 2015), and cells were monitored for 4 days by automated fluorescence microscopy. Neither the SEC cytosol fractions nor the resuspended insoluble αSyn extracts consistently caused inclusion formation in this assay, while synthetic αSyn PFFs consistently induced formation of visible inclusions (**Supplementary Figure 7C, D**). There was also no significant difference in the viability of these cells when treated with control or DLB extracts. However, even in DLB extracts with the highest relative amounts of insoluble αSyn, the extracts contained only about 50ng/ml αSyn, about 10% of the minimum required concentration of recombinant αSyn PFF (500 ng/ml) necessary to robustly induce intracellular aggregation in this experimental paradigm (data not shown).

Figures

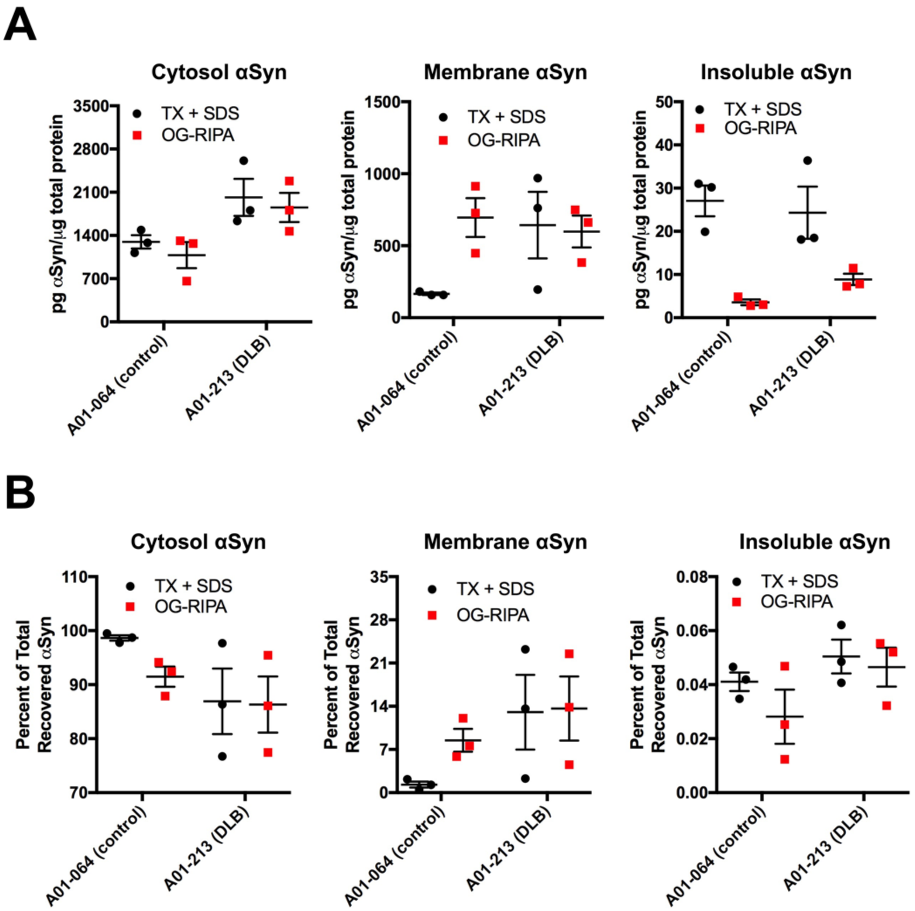

Supplementary Figure 1 – ***Comparison of OG-RIPA and 1%TX-100/2% SDS membrane fraction extraction buffers***

Three pieces of cortical gray matter tissue between 600mg and 1g were excised from brains A01-064 (non-synucleinopathy control) and A01-213 (DLB) and homogenized in TBS with protease inhibitors. Each homogenate was split into two aliquots: one was sequentially extracted in 1% Triton X-100, 2% SDS, and 8M urea/5% SDS in TBS and the other was extracted in OG-RIPA buffer and 8M urea/5% SDS in TBS. Red points indicate tissue pieces extracted using OG-RIPA buffer, and black points indicate tissue pieces extracted using 1% TX-100 buffer.

A. αSyn levels in the cytosolic, membrane-associated, and insoluble extracts normalized to total protein quantified by BCA.

B. αSyn levels in the cytosolic, membrane-associated, and insoluble extracts expressed as percent of the total amount of αSyn extracted per tissue piece by mass.

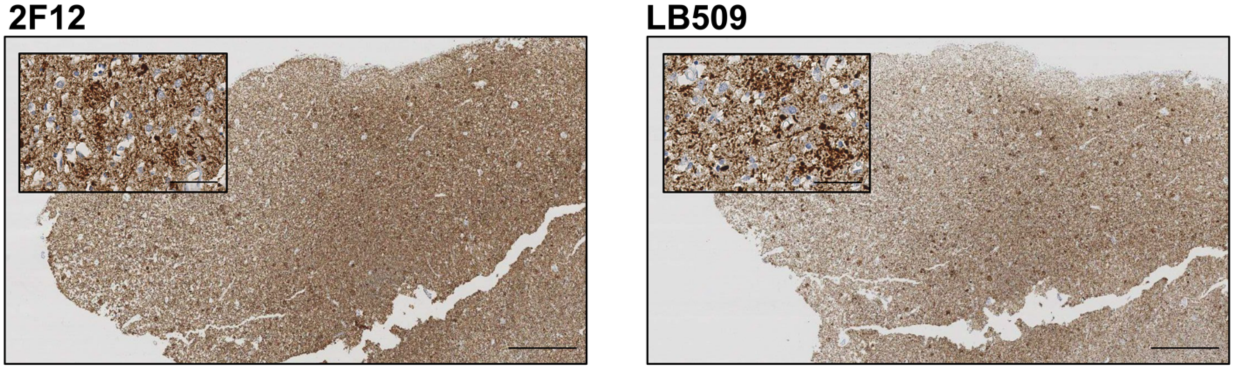

Supplementary Figure 2 – ***Comparison of LB509 and 2F12 for immunohistochemical staining of DLB cortex***

Sections from the same block of DLB brain (Syn7) were stained using 1:2000 dilutions of either 2F12 (left) or LB509 (right), another mouse monoclonal αSyn commonly used in the histopathological diagnosis of synucleinopathies. Low-objective scale bars are 500µm and inset scale bars are 50µm.

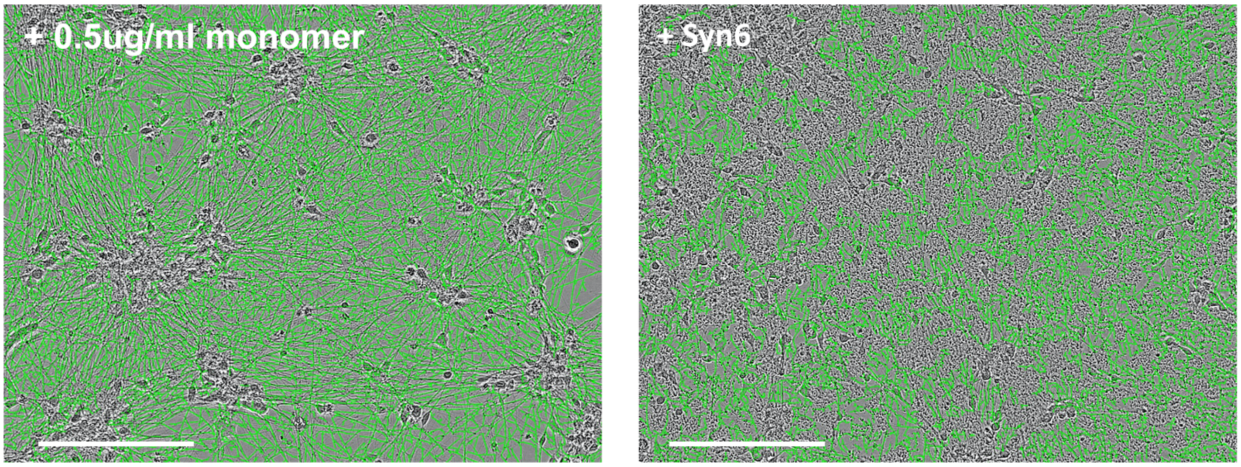

Supplementary Figure 3 – ***Masking of neurites using Incucyte image analysis software***

Incucyte analysis software, used in the neurotrack mode, applies a mask to images to identify neurites. In these representative images, neurons treated with recombinant αSyn monomer (left) show largely intact neurites, whereas neurons treated with Syn6 DLB extract display neurite retraction (right). Images were taken at 88hrs. post-treatment. Bars are 200µm.

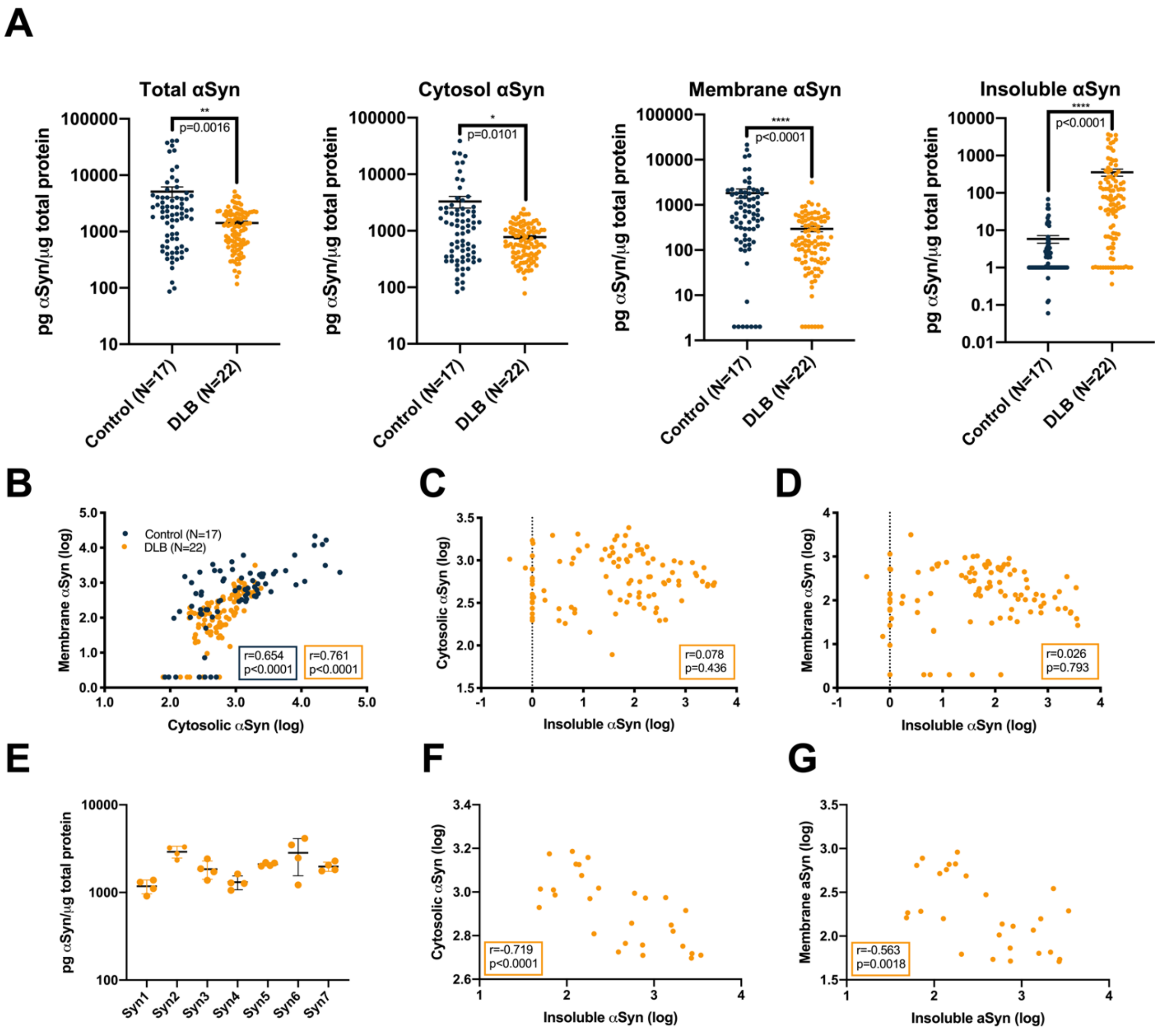

Supplementary Figure 4 – ***Biochemical extraction and subcellular fractionation of αSyn in DLB vs. synucleinopathy-free control frontal cortices, and correlations between subcellular αSyn pools***

A. Levels of αSyn in frontal cortical gray matter extracts from 17 synucleinopathy-free control brains and 22 DLB brains were calculated by normalizing ELISA-measured αSyn concentrations to BCA-measured total protein in each fraction. Mann-Whitney non-parametric comparison of ranks showed significant differences between αSyn levels of control and DLB patients in the cytosolic, membrane-associated, insoluble, and total αSyn comparisons. 3-6 tissue pieces were analyzed per brain; each point represents a single piece. Bars represent means with SEMs.

B. Correlation analysis of all frontal cortex tissue extracts revealed a strong relationship between the cytosolic and membrane fractions (control: Spearman r=0.656, p<0.0001, 95% CI: 0.494-0.770, DLB: Spearman r=0.761, p<0.0001, 95% CI: 0.662-0.834). Data were log-transformed to normalize distributions for analysis.

C-D. No correlation between cytosolic and insoluble (urea-extracted and "Lewy-associated") αSyn across all DLB frontal cortical extracts. Similar comparison of the membrane vs. insoluble fractions also showed no correlation (insoluble v. cytosol: Spearman r=-0.078, 95% CI: -0.118 to -0.267, p=0.436; insoluble v. membrane: Spearman r=-0.026, 95% CI: -0.174 to -0.224, p=0.793).

E. DLB brains from the Mayo Clinic brain bank consistently showed greater insoluble αSyn levels (compare to levels for all cases in A-far right graph). Bars represent means with SDs.

F-G. Correlation analysis between the insoluble and cytosolic or membrane fractions of Mayo Clinic cases reveals significant inverse correlations between insoluble vs. membrane and insoluble vs. cytosolic extracts (insoluble v. cytosol: Spearman r=-0.719, 95% CI: -0.864 to -0.464, p<0.0001; insoluble v. membrane: Spearman r=-0.563, 95% CI: -0.778 to -0.230, p=0.0018). Data were log-transformed to normalize distributions.

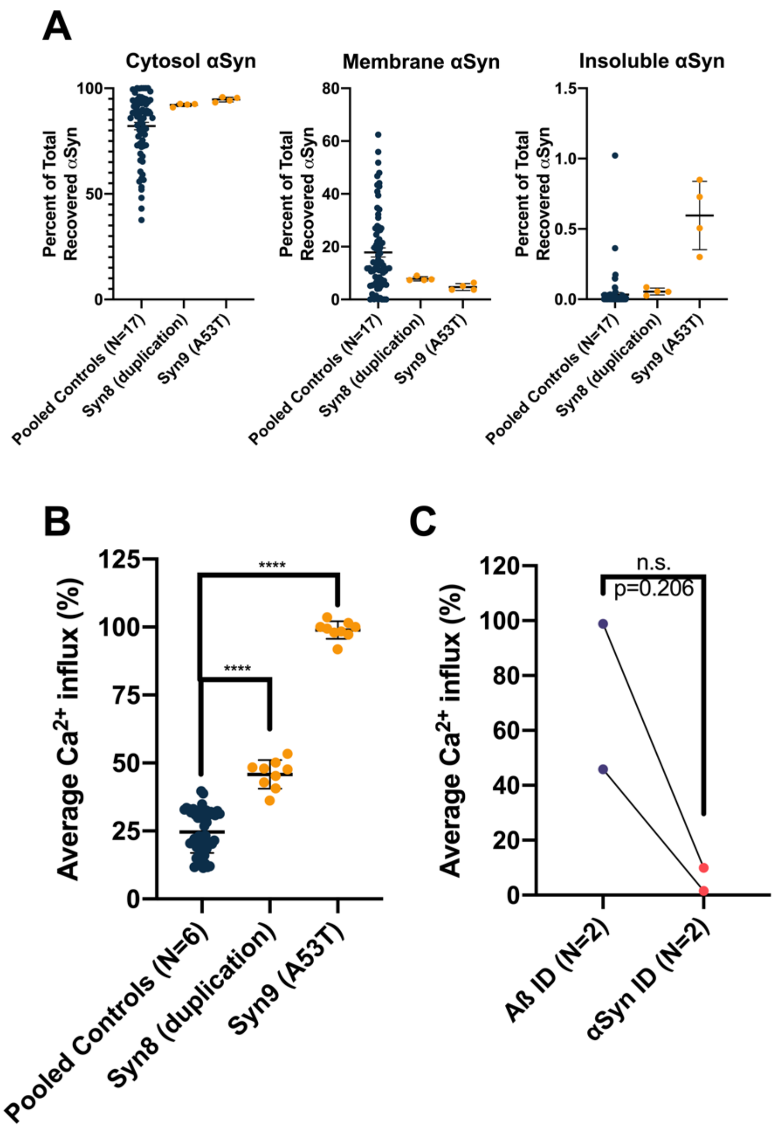

Supplementary Figure 5 – ***Analysis of SNCA duplication and A53T mutant brain cytosolic extracts***

A. Sequential extraction analysis of brain samples from patients with familial DLB showed only mild elevation of insoluble αSyn in the brain with A53T mutant αSyn expressed as a percentage of the total extracted αSyn. Interestingly, brain carrying an extra copy of the *SNCA* gene did not show elevated insoluble αSyn. For comparison, all pieces of control cortical tissue are pooled and displayed. We did not perform any statistical analyses because the discrepancies in sample size (n=74 compared to n=4). Of note, the *SNCA* duplication brain came from a male patient who died at age 63, while the A53T αSyn mutant brain came from a male patient who died at age 57.

B. Despite a low pathology burden as assessed by percentage of insoluble αSyn, both the cytosolic sample generated from the *SNCA* patient and that generated from the A53T αSyn patient increased the influx of calcium into artificial vesicles (unpaired T-tests, both p-values <0.0001). Notably, treatment of vesicles with the A53T αSyn sample lead to near-complete permeabilization. Nine technical replicates were run per cytosol.

C. As with cytosolic extracts from DLB brains with wildtype αSyn, immunoneutralization with 2F12 anti-αSyn antibody (2F12, right) reduces the DLB extract-induced permeabilization of the vesicles compared to immunoneutralization anti-Aβ antibody (4G8, left), although this effect does not reach significance in this small sample size (paired T test, p=0.206). For each patient sample, nine technical replicates were used to generate the data point. Each pair of points connected by a line represents a single cytosolic HMW extract.

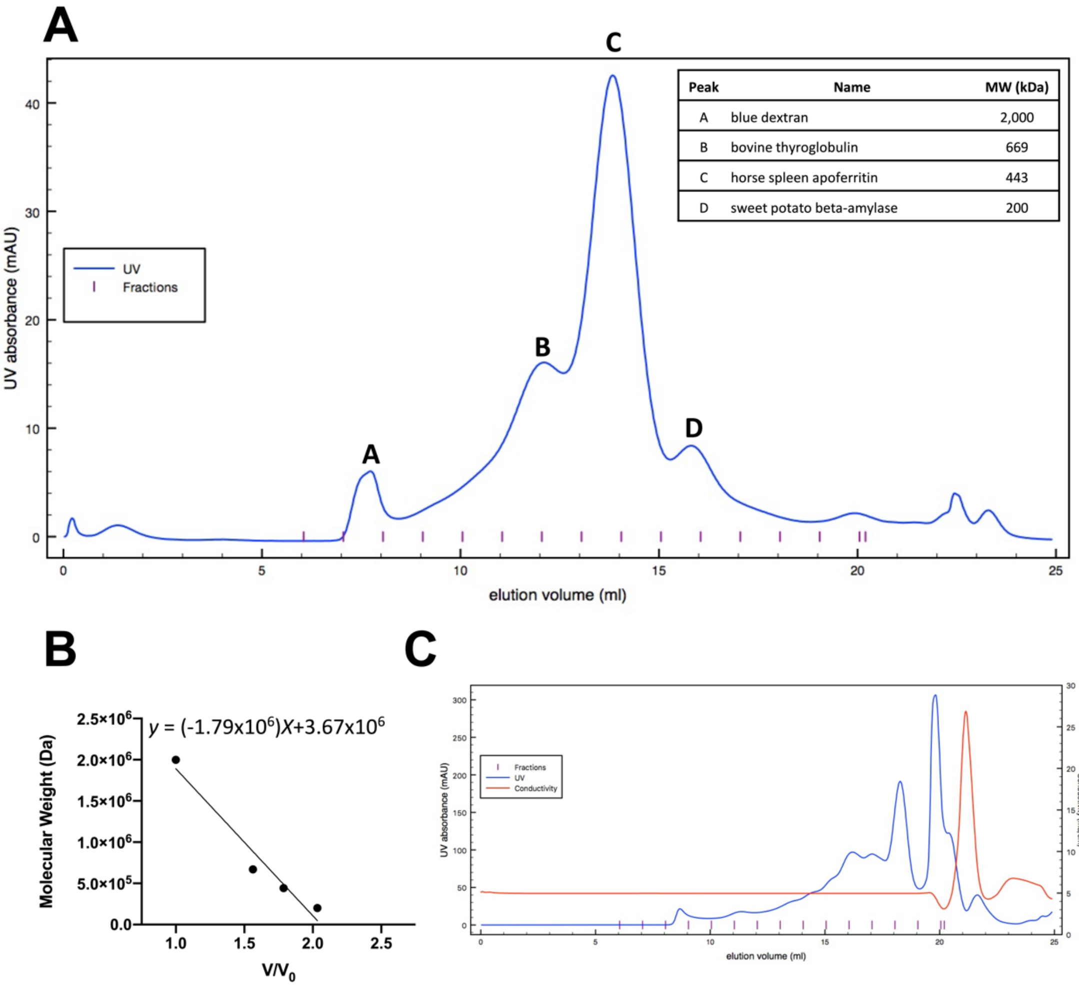

Supplementary Figure 6 – ***Establishment of standard molecular weight curve for SEC elution volumes***

A. A known mixture of proteins, identified in the table inset, were run through a Superose 6 10/300 SEC column. UV peaks corresponding to the elution of these standards (labeled A-E) were recorded. Peak A, corresponding to the elution of blue dextran, indicates the void volume, or the volume at which molecules too large to be slowed by the gel matrix elute.

B. Using the ratio of elution volume to void volume (*V/V_0*), an exponential curve was fit to the standards and used for future calculation of molecular weight of proteins based on elution volumes. This curve indicates the high resolution of the Superose 6 column between 2,000 and 200kDa and its poor resolution at lower molecular weights.

C. A cytosolic extract of control brain MGH 1901 shows a typical elution profile. Conductivity (red) is used to indicate the completion of the run, as the late peak indicates the elution of small buffer molecules.

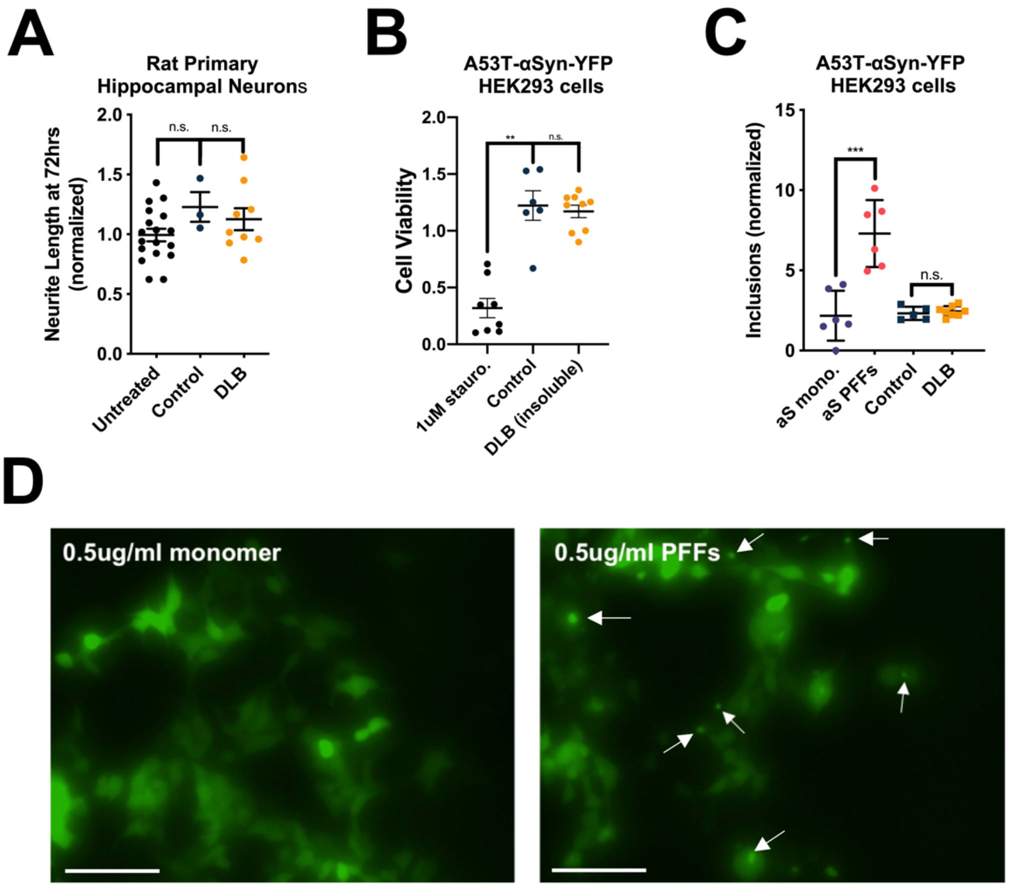

Supplementary Figure 7 – *Bioactivity of soluble and insoluble extracts of frontal cortex*

Pooled HMW SEC fractions and insoluble fractions (either urea-solubilized or sonicated in culture medium) were analyzed for bioactivity in cellular assays.

A. Rat primary hippocampal neurons were treated with pooled HMW SEC fractions in triplicate for 72 hr. Neurite morphology was assessed using the Incucyte live microscopy imaging platform. No differences in neurite length (means with SEMs) were detected between control and DLB fractions. All SEC fraction treatment groups trended towards an increase in mean neurite length compared to untreated cells. Neurons were treated with extracts from a single control brain and 3 different DLB brains. Each point represents a single sample, which was tested in 3 technical replicates randomly assigned to non-adjacent wells on the plate.

B. HEK293 cells expressing a A53T-αSyn-YFP fusion construct were proteofected in triplicate wells with sonication-solubilized brain material or synthetic PFF in cell growth medium. 3 DLB brain extracts and 2 non-synucleinopathy control brain extracts were analyzed. After a treatment

period of 96 hours, cell viability was assessed using the CellTiter Blue assay. There was a significant difference between viability of cells treated with staurosporin and cells treated with extracts from 2 non-synucleinopathy control subjects (Mann-Whitney comparison of ranks, two-tailed p=0.0013). There was no significant difference in viability between cells treated with extracts from 3 DLB patient brains compared to the non-synucleinopathy control extracts (unpaired T-test, two-tailed p=0.864). Cell viability was normalized to triplicate wells treated with proteofection agent alone.

C-D. Inclusion formation was assessed and quantified using the Incucyte live fluorescent imaging and analysis platform. Each inclusion count was normalized to the total YFP area per image to control for cell density in a given field. An unpaired T-test comparing inclusion formation in cells treated with 0.5µg/ml recombinant αSyn monomer or PFFs showed a significant difference of means (two-tailed p=0.0007), while a similar analysis comparing inclusion formation in cells treated either with non-synucleinopathy control or DLB brain extracts did not show a significant difference (two-tailed p=0.550). Means and standard errors are shown. Scale bars are 100µm.
